# Diagnosis of Sinonasal Carcinoma in the Emergency Department: A Case Report Highlighting Red Flag Symptoms

**DOI:** 10.5070/M5.52257

**Published:** 2026-04-30

**Authors:** Daniela Usuga, Ayomide Osunjima, Colin Danko

**Affiliations:** * University of Texas Southwestern Medical Center, Department of Emergency Medicine, Dallas, TX; ^ University of Texas Southwestern Medical Center, School of Medicine, Dallas, TX

## Abstract

Sinonasal carcinoma is a rare malignancy that often presents with nonspecific symptoms, making early diagnosis challenging. Delayed recognition can lead to disease progression and worse outcomes. This case report emphasizes the importance of early identification, prompt imaging, and multidisciplinary management. A 73-year-old male with a history of anemia and hypertension presented with progressive facial swelling, 20-pound weight loss, and vision loss in the left eye over two months. Initially misdiagnosed with bacterial sinusitis, his symptoms persisted. Examination revealed a proptotic left eye and a large obstructive nasal mass. Computed tomography (CT) and MRI (magnetic resonance imaging) demonstrated a destructive sinonasal mass with skull base and intracranial extension. A biopsy confirmed sinonasal carcinoma, and the patient was started on chemoradiation therapy due to the inoperability of the tumor.

This case underscores the importance of recognizing red flag symptoms such as persistent facial swelling and neurological deficits. Multimodal imaging played a critical role in diagnosis. The literature suggests sinonasal carcinoma is often diagnosed late, reinforcing the need for early suspicion and specialist referral. Sinonasal carcinoma should be considered in patients with persistent facial or nasal symptoms. Timely imaging, biopsy, and interdisciplinary care are essential for optimizing outcomes.

**Topics:**

Facial masses, sinonasal carcinoma, nasal masses, facial swelling.

**Figure:**
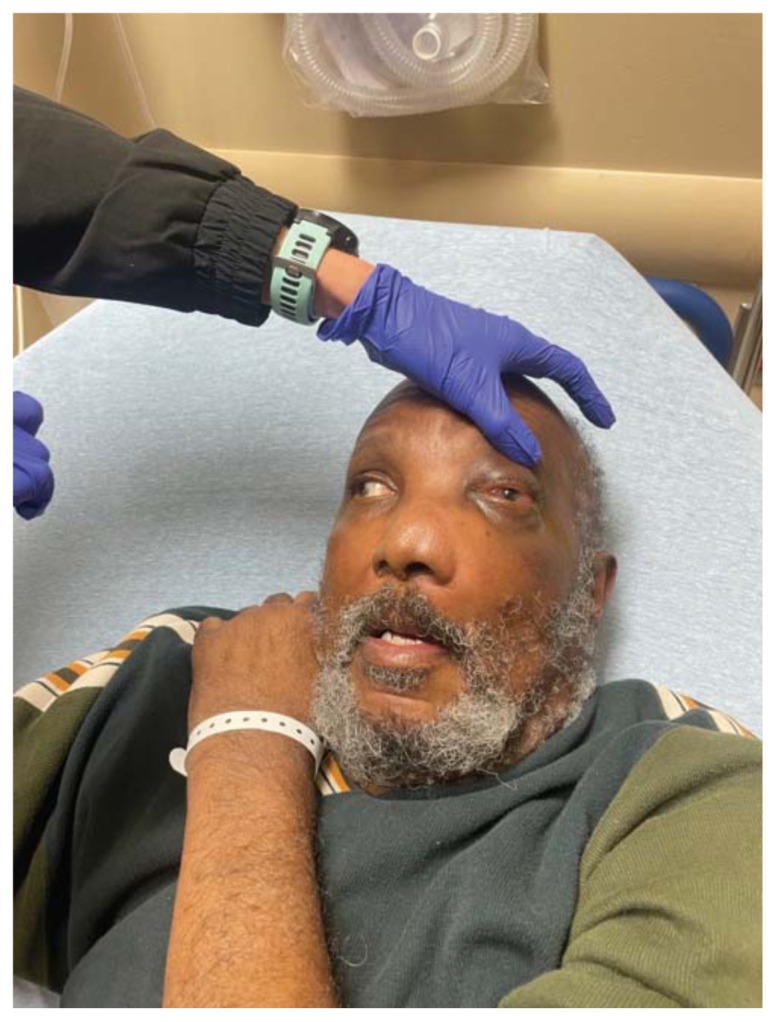


**Figure:**
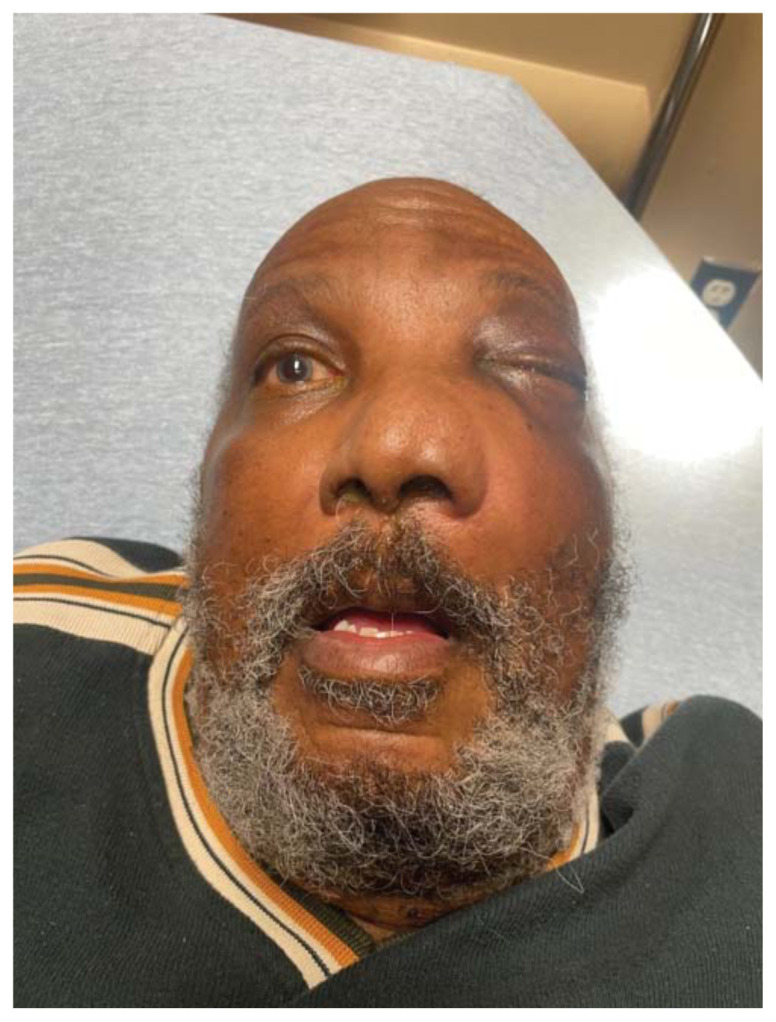


**Figure:**
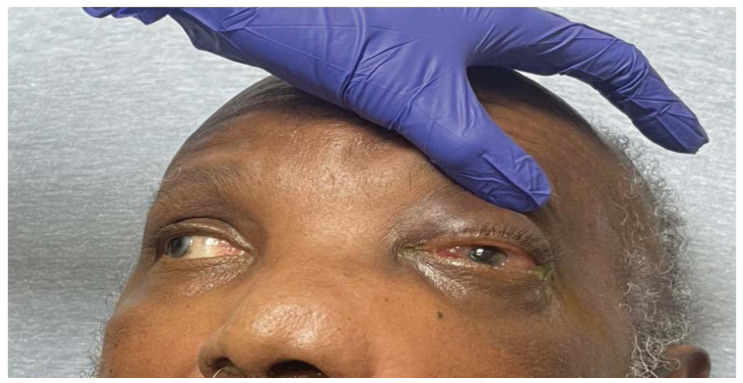


**Figure:**
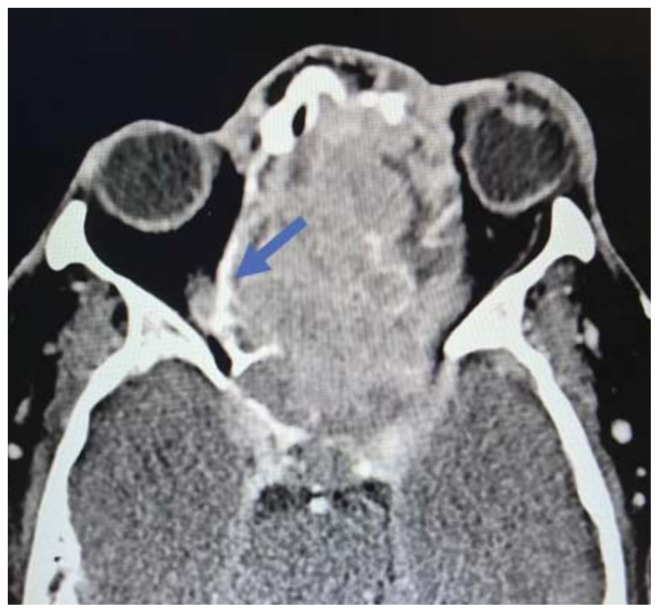


**Figure:**
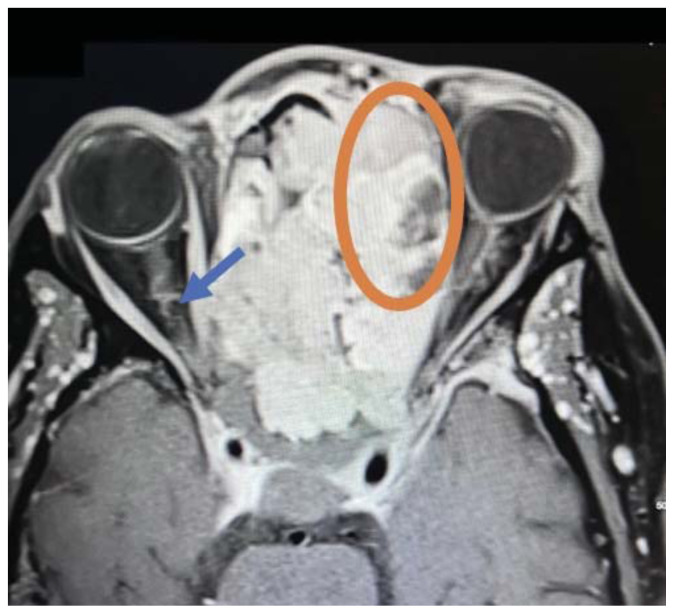


**Figure:**
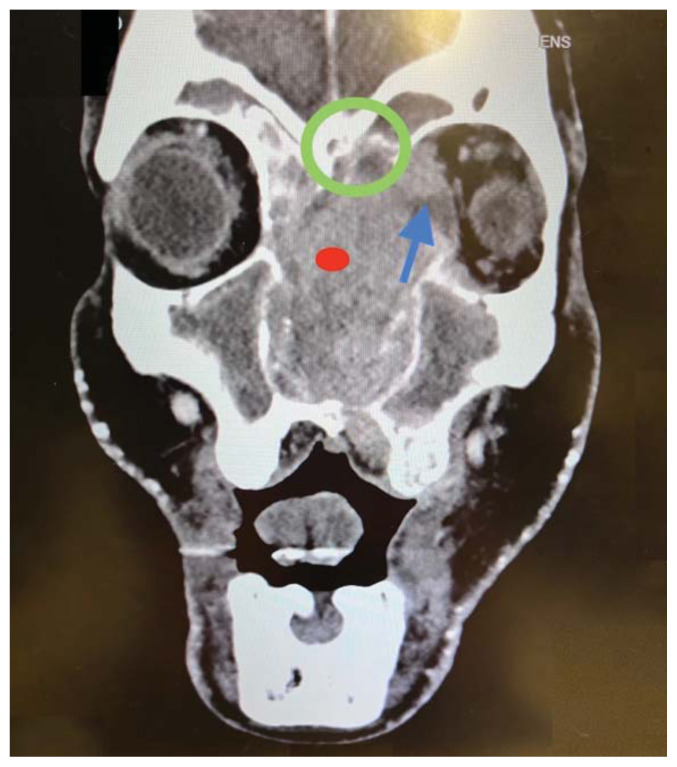


## Brief introduction

Diagnosing facial malignancies in the emergency department (ED) is challenging because they can often initially present with benign appearing symptoms suggestive of less dangerous pathology. However, catching these malignancies early in the ED can be critical to preventing disease progression and improving outcomes. A facial mass persisting for more than two weeks without signs of infection should raise suspicion for malignancy, particularly when associated with symptoms such as difficulty or painful swallowing, ear pain, voice changes, nasal blockage, epistaxis, or unexplained weight loss.^1^ Physical examination findings that heighten concern for malignant mass include tissue fixation, a firm consistency, a size exceeding 1.5 cm, or ulceration.^2^ Furthermore, oropharyngeal asymmetry, restricted jaw movement, unilateral facial swelling, and limited tongue mobility warrant close evaluation.^2^,^3^ Although unilateral upper and lower facial droop is often considered a hallmark feature of Bell’s palsy, maintaining a broad differential diagnosis is crucial because this can also indicate an underlying malignancy compressing cranial nerves.^2^,^4^,^5^–^10^

This case report emphasizes the need for timely recognition of these clinical signs in the ED to facilitate early diagnosis and improve patient outcomes. Written consent was obtained for publication.

## Presenting concerns and clinical findings

A 73-year-old male with a past medical history of anemia, hypertension, bilateral inguinal hernias complicated by bowel obstructions, and benign prostatic hyperplasia presented to the ED due to facial pain and swelling for the past two months. The patient reported that he initially presented to an urgent care one month prior due to facial swelling with associated pain and congestion, and he was given antibiotics and Afrin nasal spray for suspected bacterial sinusitis. Despite this treatment, the swelling and pain significantly worsened over the next few weeks. He eventually noticed worsening vision in his left eye and presented it to an outside ENT clinic who sent the patient to the ED because they were concerned for a mass. Upon further assessment in the ED, the patient additionally reported a chronic greenish-yellow nasal discharge with intermittent epistaxis, discomfort with swallowing, and a reported 20-pound weight loss over the previous month. He denied experiencing headache, ear pain, vertigo, hoarseness, fever, chills, nausea, vomiting, or shortness of breath. Initial differentials for this case included chronic sinusitis, inverted papilloma, idiopathic angioedema, facial malignancies, cavernous sinus thrombosis, and superior vena cava (SVC) syndrome.

## Significant findings

Physical exam revealed a gross deformity of the left side of the face with soft tissue swelling with no overlying skin changes. The left eye was proptotic and completely immobile, with cranial nerve (CN) 3, 4, and 6 palsies. Additionally, a large obstructive mass was noted in the left naris with resultant rightward displacement of the nasal septum. Intraocular pressures (IOPs) were measured to be 6 mmHg in the right eye and 11 mmHg in the left eye.

Imaging, including maxillofacial and neck soft tissue CTs revealed a large, destructive, soft tissue mass centered in the nasal cavity with significant osseous destruction of the midface and skull base (red highlighted area). There was mild intracranial extension with the mass abutting or infiltrating the inferior frontal lobes (green highlighted area). The nasal cavity and nasopharynx were obstructed (orange highlighted area). The mass invaded the left orbit with associated left-sided proptosis, left globe deformity, and compressed the left optic nerve and the left optic chiasm (blue arrows).

Ophthalmology and otorhinolaryngology were consulted and recommended more imaging for further evaluation. Differentials for this mass included primary nasopharyngeal carcinoma, esthesioneuroblastoma, atypical aggressive meningioma, pituitary adenoma and metastasis. The plan was to admit the patient for a biopsy of the mass and pain control.

## Patient course

The patient was admitted to the hospital and underwent an MRI which showed a large, destructive, enhancing soft tissue mass primarily centered in the left nasal cavity. Further imaging revealed involvement and inferior displacement of the soft palate with narrowing of the posterior oral cavity. The mass contacted the left optic nerve with encasement of the orbital and canalicular segments. The mass was inseparable from the cribriform plate and had intracranial extension.

A biopsy of the nasal cavity mass was performed which confirmed the diagnosis of sinonasal carcinoma. The patient was started on dexamethasone 4 mg IV every six hours for vasogenic edema. Due to parenchymal brain involvement rendering resection unfeasible, radiation oncology was involved. Given the rapidly worsening nasal mass with mass effect and the overall complexity of this case, the tumor board determined that radiation therapy should be initiated, and the patient was consented for chemoradiation therapy.

He tolerated the first round of chemotherapy well without any significant side effects, although he did continue to have significant facial pain which was managed with oral pain medications. He was subsequently discharged home with follow up appointments set with oncology and radiation oncology for continuation of chemotherapy and radiation therapy. Patient followed up two weeks later with radiation oncology for placement of an implantable infusion port for chemotherapy. During this visit patient had right upper extremity swelling, concerning for a deep vein thrombosis, and patient was recommended to go to the ED to work up this swelling. Patient did not present to ED despite recommendation. Patient was lost to follow up, and family members report that patient started having erratic and aggressive behavior at home with periods of disappearing. Patient appeared at an outside ED, where he was found sleeping outside in the rain and had a chief complaint of being cold and needing to find a place to live. Patient left against medical advice (AMA) because we refused to get any workup at this time. Two months later, patient presented to outside hospital in shock requiring four pressors, empiric antibiotics, and fluids. Patient’s wife made patient a do not resuscitate (DNR), and the decision was made to not further escalate care, leading to the patient’s eventual death.

## Discussion

This case report highlights the importance of early recognition and intervention in facial malignancies, particularly in the ED. The case underscores the challenge of differentiating malignant masses from benign conditions, emphasizing the role of a thorough history, physical examination, and advanced imaging in timely diagnosis. The primary strengths of this report include its detailed clinical course, multimodal imaging findings, and interdisciplinary approach to patient care. Furthermore, is important to have a wide differential when a patient presents with facial swelling, considering not only anaphylaxis but also other potential causes such as chronic sinusitis, inverted papilloma, idiopathic angioedema, facial malignancies, cavernous sinus thrombosis, and superior vena cava (SVC) syndrome.

The inclusion of multiple imaging modalities—CT, MRI, and physical exam findings—provides a robust foundation for discussing the sensitivity and specificity of these diagnostic tools in identifying sinonasal carcinoma.^9^–^13^ Additionally, the case highlights the value of interdisciplinary collaboration, including emergency medicine, otolaryngology, ophthalmology, oncology, and radiation oncology, in optimizing patient outcomes. However, there are limitations given the aggressive nature of the malignancy. It is important to recognize red flag symptoms including vision loss, unilateral proptosis, nasal obstruction, and weight loss further supporting the need for urgent biopsy.^9^–^13^ While imaging findings were highly suggestive of malignancy, the lack of initial biopsy or referral contributed to disease progression before definitive diagnosis and treatment. Additionally, although this case illustrates a common presentation of sinonasal carcinoma, its applicability to other malignancies with varied presentations may be limited.

The imaging findings in this case—osseous destruction, intracranial extension, orbital invasion, and mass effect—strongly suggest malignancy, consistent with prior literature on sinonasal carcinoma. Studies indicate that CT imaging has high sensitivity (above 90%) for detecting bone invasion, whereas MRI is superior for assessing soft tissue involvement and perineural spread.^8^

Facial malignancies, including sinonasal carcinomas, often present with nonspecific symptoms leading to diagnostic delays.^4^–^8^ Literature suggests that persistent symptoms such as facial swelling, nasal obstruction, and cranial nerve deficits should prompt further investigation.^4^–^8^ Delays in diagnosis are associated with worse prognosis due to advanced-stage detection. This case reinforces existing research advocating for early specialist referral and biopsy when malignancy is suspected based on persistent or progressive symptoms. It additionally underscores the need for heightened vigilance in the ED for facial malignancies, emphasizing early diagnosis, multidisciplinary care, and the role of imaging in guiding clinical decision-making.

## Supplementary Information


















