# Supplementary figures and images for: Diagnosis of Sinonasal Carcinoma in the Emergency Department: A Case Report Highlighting Red Flag Symptoms
Source: J Educ Teach Emerg Med. 2026 Apr 30;11(2):V35–40. doi: 10.5070/M5.52257 (PMC13152384; doi:10.5070/M5.52257)

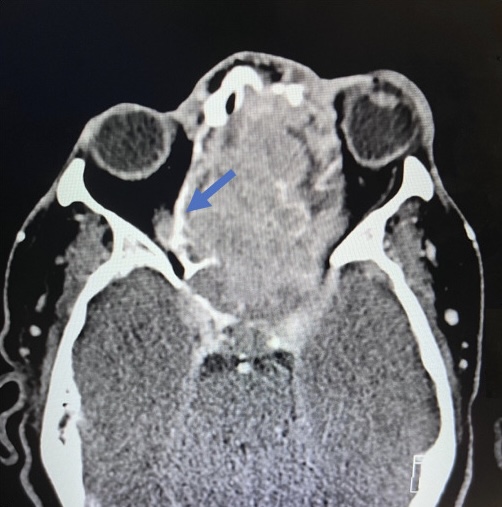

Supplement: Supplementary file 1 [file 11-2-V35-Supp1.jpg]

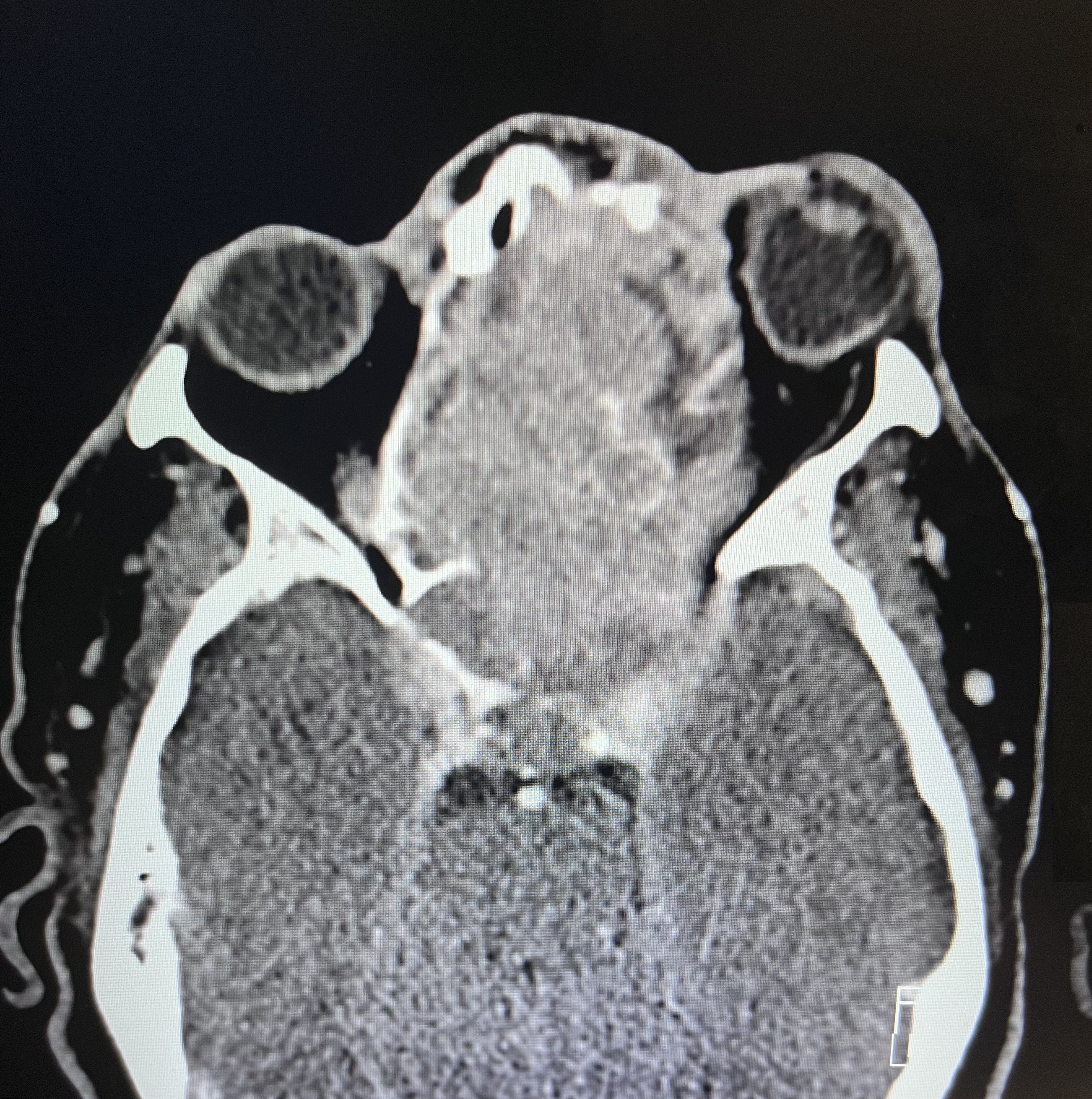

Supplement: Supplementary file 2 [file 11-2-V35-Supp2.jpg]

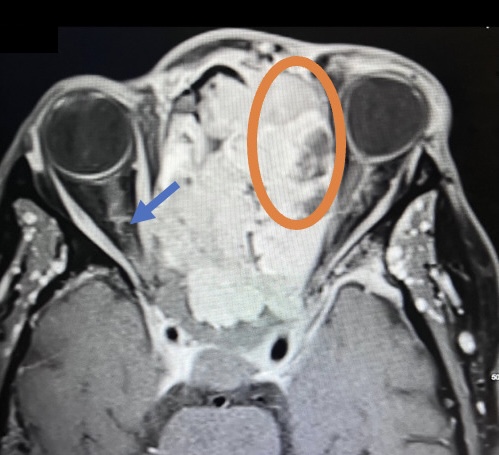

Supplement: Supplementary file 3 [file 11-2-V35-Supp3.jpg]

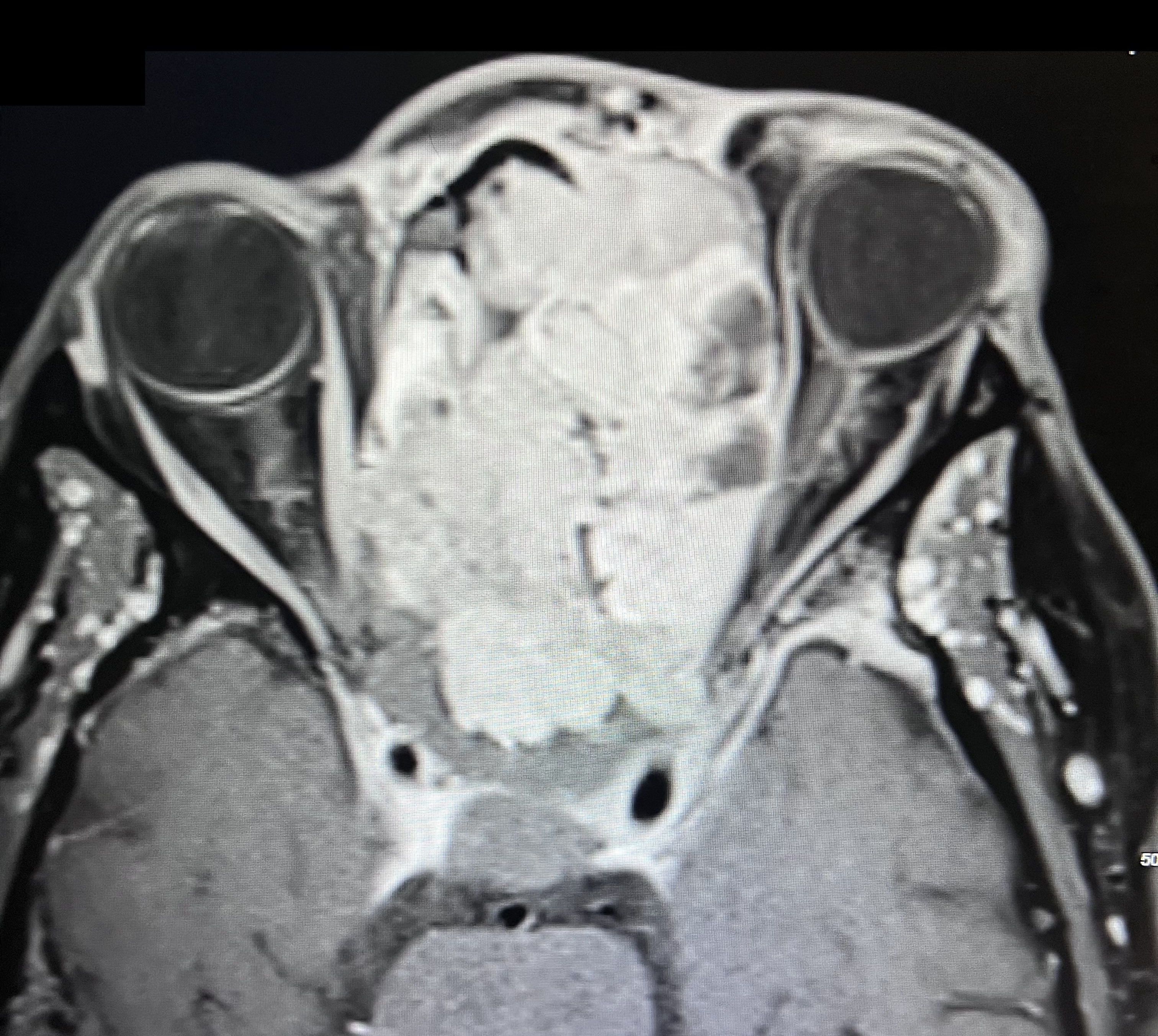

Supplement: Supplementary file 4 [file 11-2-V35-Supp4.jpg]

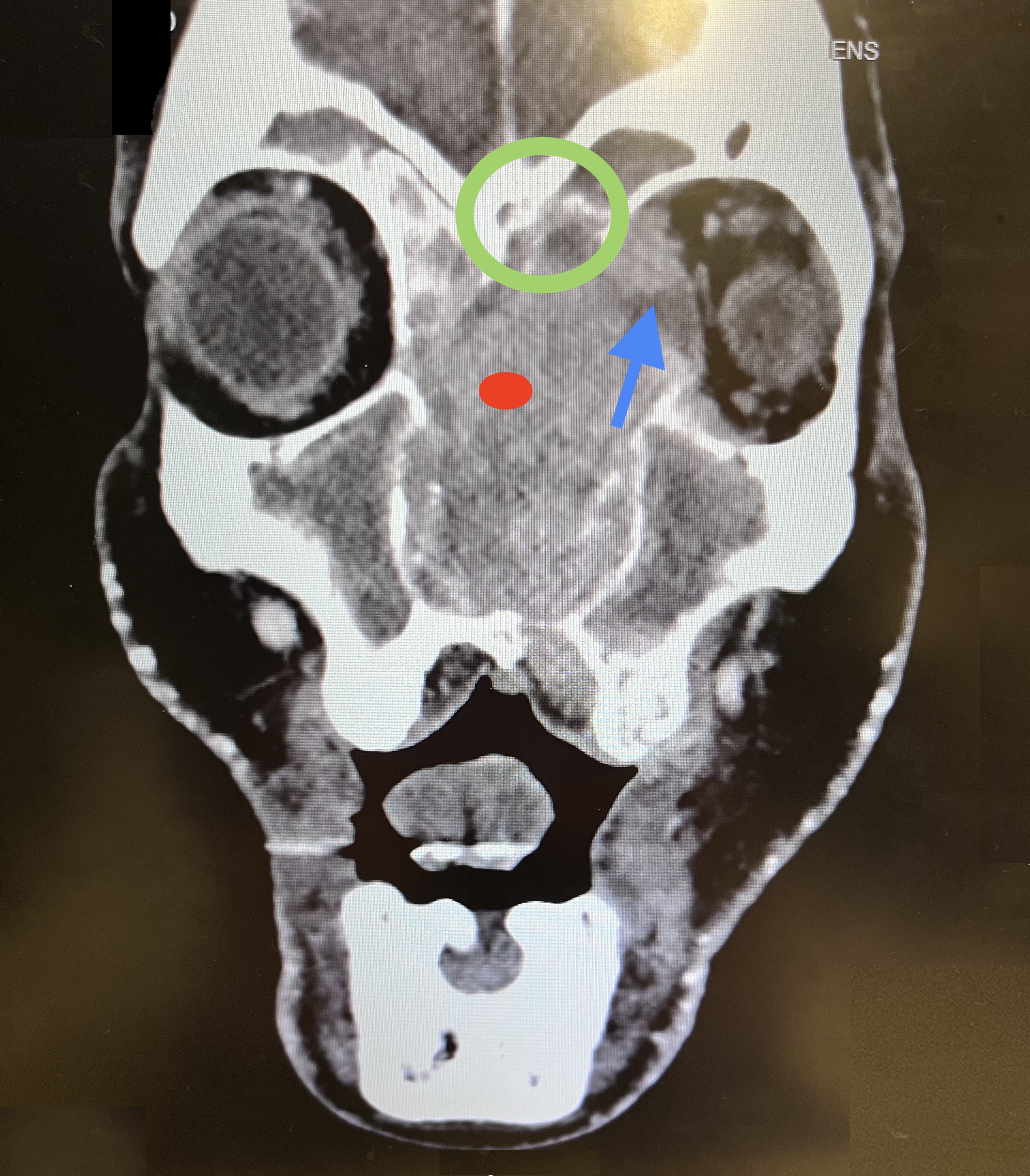

Supplement: Supplementary file 5 [file 11-2-V35-Supp5.jpeg]

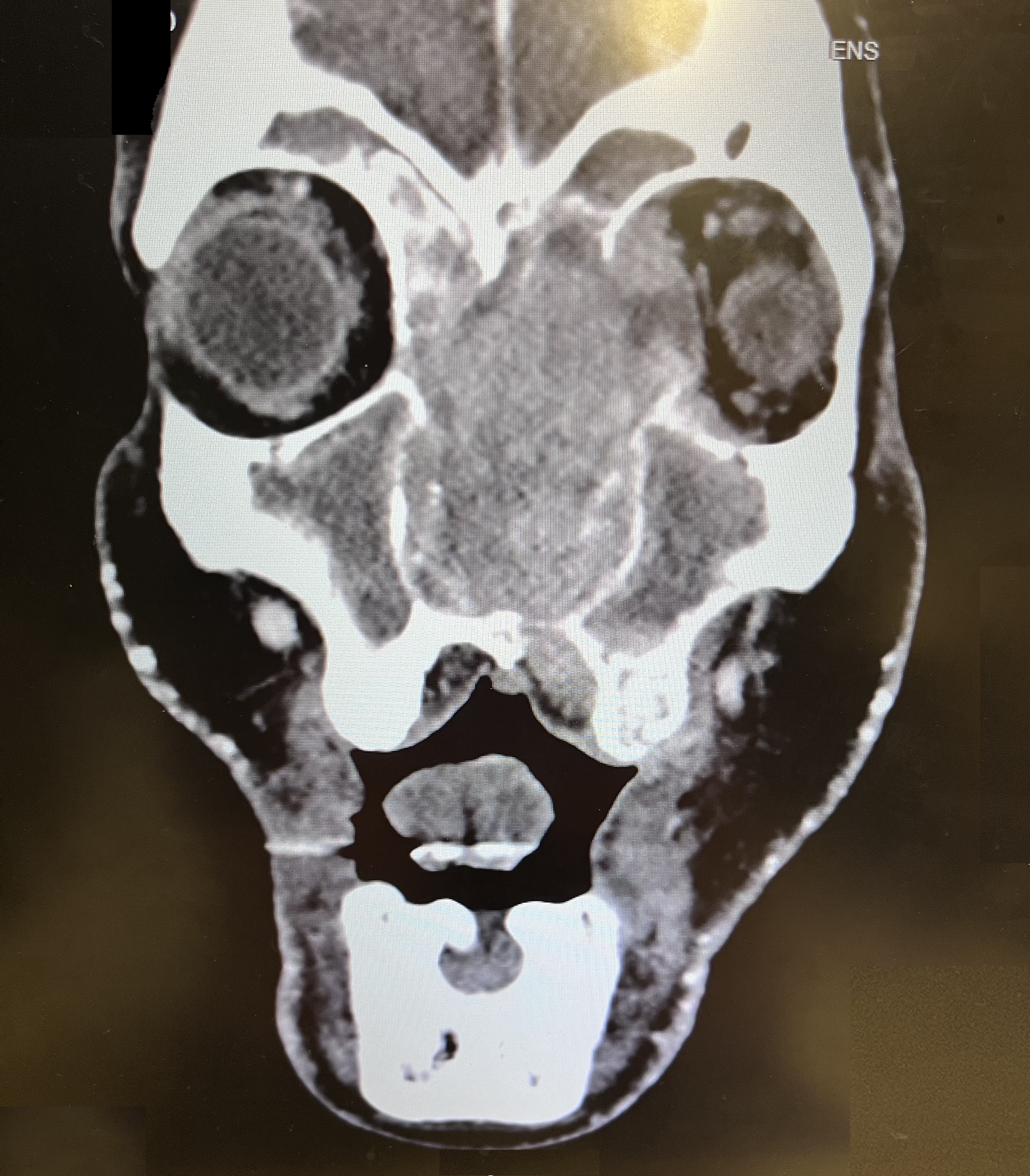

Supplement: Supplementary file 6 [file 11-2-V35-Supp6.jpeg]

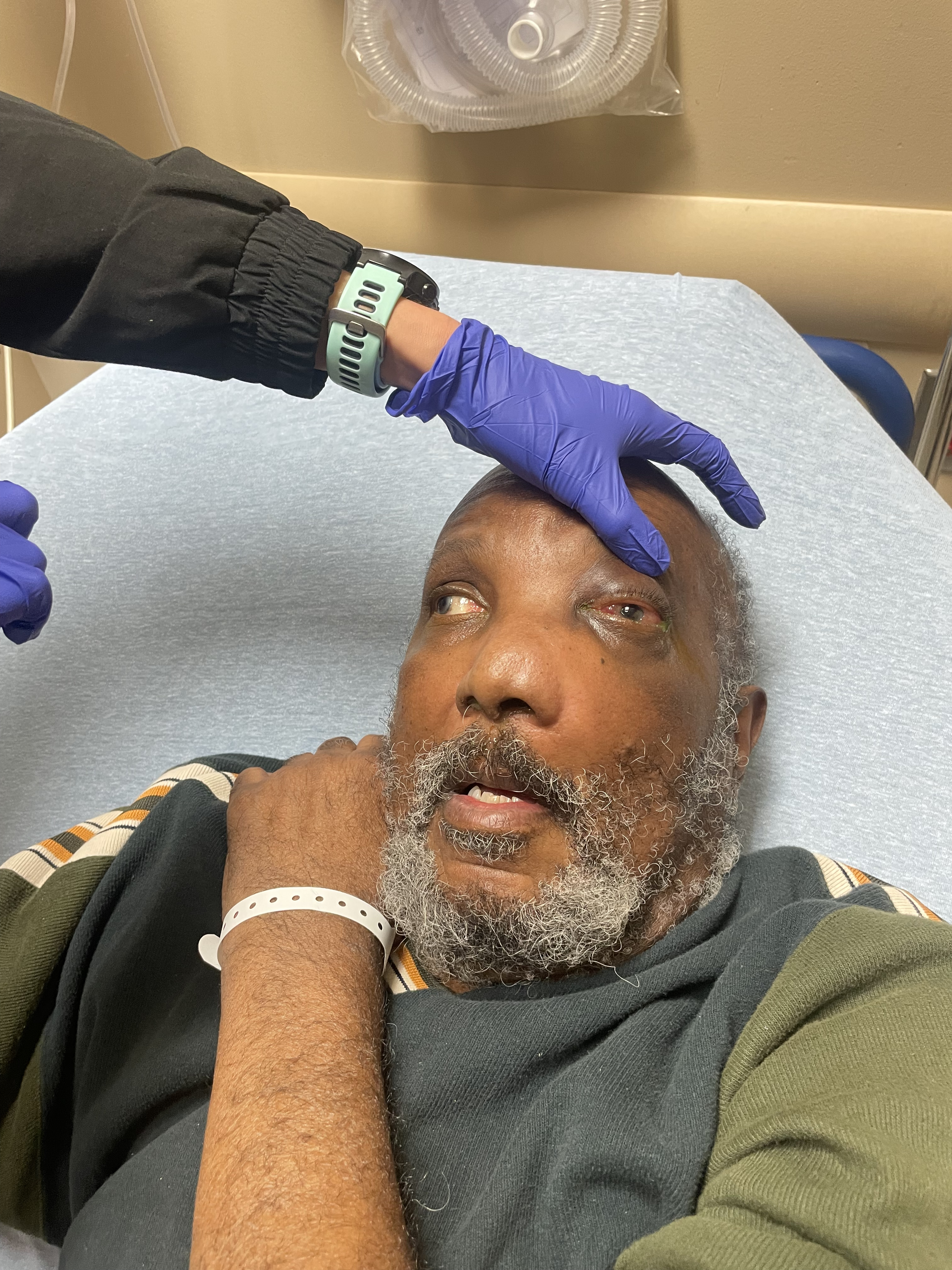

Supplement: Supplementary file 7 [file 11-2-V35-Supp7.jpeg]

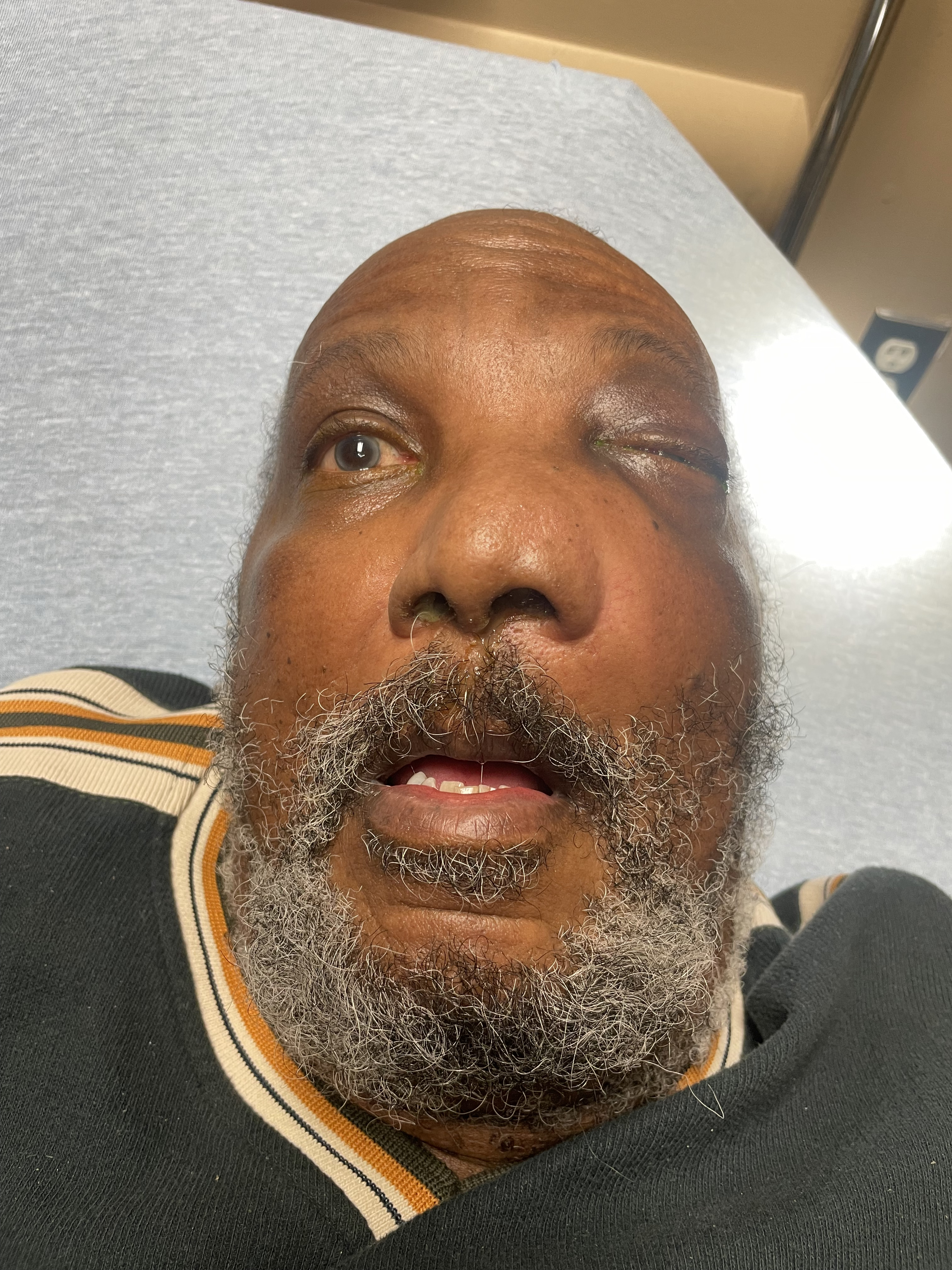

Supplement: Supplementary file 8 [file 11-2-V35-Supp8.jpg]

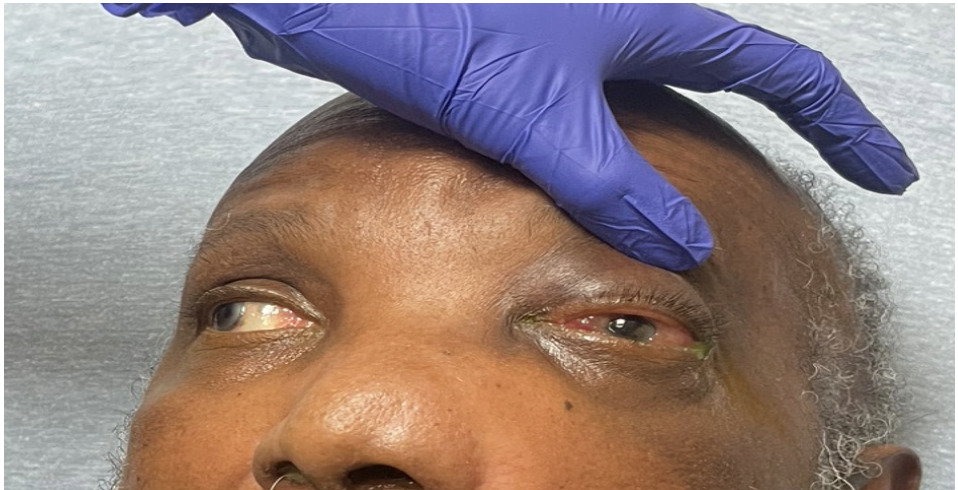

Supplement: Supplementary file 9 [file 11-2-V35-Supp9.jpg]
